# Nature-Inspired Multi-Level Thresholding Integrated with CNN for Accurate COVID-19 and Lung Disease Classification in Chest X-Ray Images

**DOI:** 10.3390/diagnostics15121500

**Published:** 2025-06-12

**Authors:** Wafa Gtifa, Ayoub Mhaouch, Nasser Alsharif, Turke Althobaiti, Anis Sakly

**Affiliations:** 1 Laboratory of Automation and Electrical Systems and Environment, Monastir National School of Engineers (ENIM), University of Monastir, Monastir 5035, Tunisia; sakly_anis@yahoo.fr; 2 Laboratory of Electronic and Microelectronic, Faculty of Sciences of Monastir, University of Monastir, Monastir 5035, Tunisia; ayoubmhaouch46@gmail.com; 3 Department of Sciences and Technology, Ranyah University College, Taif University, Taif 21944, Saudi Arabia; n.alsharif@tu.edu.sa; 4 Department of Computer Science, Faculty of Science, Northern Border University, Arar 73222, Saudi Arabia; turke.althobaiti@nbu.edu.sa

**Keywords:** chest X-ray classification, multi-level thresholding, animal migration optimization, electromagnetism-like optimization, harmony search algorithm, convolutional neural network, COVID-19 diagnosis

## Abstract

**Background/Objectives**: Accurate classification of COVID-19 from chest X-rays is critical but remains limited by overlapping features with other lung diseases and the suboptimal performance of current methods. This study addresses the diagnostic gap by introducing a novel hybrid framework for precise segmentation and classification of lung conditions. **Methods**: The approach combines multi-level thresholding with the advanced metaheuristic optimization algorithms animal migration optimization (AMO), electromagnetism-like optimization (EMO), and the harmony search algorithm (HSA) to enhance image segmentation. A convolutional neural network (CNN) is then employed to classify segmented images into COVID-19, viral pneumonia, or normal categories. **Results**: The proposed method achieved high diagnostic performance, with 99% accuracy, 99% sensitivity, and 99.5% specificity, confirming its robustness and effectiveness in clinical image classification tasks. **Conclusions**: This study offers a novel and technically integrated solution for the automated diagnosis of COVID-19 and related lung conditions. The method’s high accuracy and computational efficiency demonstrate its potential for real-world deployment in medical diagnostics.

## 1. Introduction

The COVID-19 pandemic continues to exert a profound and lasting influence on the global landscape, posing unprecedented challenges to public health systems worldwide. As of January 2024, the World Health Organization reported that SARS-CoV-2 variants have spread to 180 countries, with cumulative cases surpassing 770 million and deaths exceeding 7 million [1]. The virus’s transmission dynamics and mortality rates far outpace those of previous viral outbreaks, as evidenced by comprehensive epidemiological studies [2,3]. These staggering figures underscore the urgent need for effective diagnostic solutions to mitigate the pandemic’s ongoing impact, particularly considering the emergence of new variants and the persistent burden on healthcare systems [4,5].

The surge in demand for accurate and accessible diagnostic strategies has exposed significant limitations in existing approaches. While RT-PCR remains the gold standard, recent studies highlight critical shortcomings, including false negative rates of 15–20% during early infection stages, prolonged processing times of 24–48 h, and high operational costs [6,7]. Point-of-care (POC) tests, though convenient, exhibit inconsistent performance, with sensitivity and specificity ranging from 65–95% and 70–99%, respectively, across viral variants [8]. These challenges are further exacerbated in resource-limited settings where reagent shortages, equipment maintenance issues, and logistical constraints hinder diagnostic capacity [9,10]. Moreover, the economic and social impacts of delayed or inaccurate diagnoses have been well documented, with studies estimating billions of dollars in healthcare costs and productivity losses [11,12].

In response to the growing demand for efficient COVID-19 diagnostics, emerging technologies are reshaping the diagnostic landscape. Artificial intelligence (AI) has emerged as a transformative tool [13], with deep learning algorithms achieving detection accuracies exceeding 95% in clinical trials [14]. Among advanced imaging techniques, chest X-rays have gained significant attention due to their accessibility, cost-effectiveness, and rapid imaging capabilities. When integrated with AI-driven analysis, X-ray imaging has demonstrated remarkable diagnostic potential. For instance, AI-enhanced X-ray analysis has been shown to identify COVID-19-specific patterns with high accuracy, outperforming traditional radiological methods [15]. Moreover, machine learning algorithms can differentiate COVID-19 pneumonia from other respiratory conditions with 94% accuracy while reducing interpretation time by 60% [16,17]. These advancements are particularly critical considering the ongoing emergence of new variants and the need for scalable, cost-effective diagnostic solutions [5].

This research presents a novel approach to segmenting and classifying lung X-ray images to distinguish between COVID-19, viral pneumonia, and normal lung conditions. The primary objective is to develop a robust and automated method for detecting lung abnormalities, with a focus on enhancing the accuracy and efficiency of early diagnosis. By leveraging advanced image segmentation techniques optimized by state-of-the-art algorithms such as AMO, EMO, and HSA and employing a CNN for classification, this study aims to provide a reliable tool for clinical decision making in respiratory health care.

The motivation behind this work stems from the urgent need to address the limitations of current diagnostic approaches, particularly in the context of emerging infectious diseases. The ability to accurately and swiftly identify COVID-19 cases is crucial for controlling outbreaks, optimizing treatment strategies, and reducing the burden on healthcare systems. By combining advanced optimization algorithms with deep learning techniques, this study seeks to bridge the gap between traditional diagnostic methods and cutting-edge technological solutions, offering a scalable and reliable tool for respiratory health care.

The proposed approach demonstrates outstanding performance, achieving up to 99% accuracy, 99% sensitivity, and 99.5% specificity across multiple evaluation metrics. These results highlight the model’s robustness in differentiating between COVID-19, pneumonia, and normal lung conditions, underscoring its potential for real-world clinical applications. While the algorithms used in this study, EMO, AMO, and HSA, are existing methods, the novelty of this work lies in their application to lung X-ray images for COVID-19 detection. This specific application has not been extensively explored in prior work and our findings confirm the effectiveness of these methods in accurately detecting lung abnormalities, particularly for distinguishing COVID-19 from other lung diseases. This approach holds significant promises for enhancing early diagnosis and supporting clinical decision making in respiratory health care, ultimately contributing to improved patient outcomes and pandemic preparedness.

Although several AI-based approaches have been proposed for COVID-19 diagnosis, many rely on pre-segmented or pre-processed chest X-ray datasets, limiting their applicability in real-time clinical environments. Furthermore, most existing segmentation techniques are either manual or based on conventional thresholding without optimization, which compromises both precision and consistency. This study addresses these gaps by proposing a fully automated diagnostic pipeline that integrates metaheuristic-optimized multi-level thresholding with CNN-based classification. The novelty lies in the application of three distinct optimization algorithms, AMO, EMO, and HSA, to enhance segmentation quality directly on raw chest X-ray images. This optimization-driven segmentation improves the clarity of lung structures, enabling more reliable classification.

This study is designed as an original experimental investigation and not as a literature review. Its primary aim is to implement and evaluate a novel, end-to-end framework for lung disease detection using optimized thresholding and machine learning. While brief background context is included for completeness, the focus remains on the integration of Otsu’s thresholding function with nature-inspired optimizers to generate high-quality segmentation masks, which are then used to train a custom convolutional neural network. The proposed method demonstrates improved diagnostic performance across multiple classes, COVID-19, pneumonia, and normal lungs, making it a scalable and practical solution for deployment in both advanced and resource-constrained healthcare systems.

## 2. Literature Review

The diagnosis of lung diseases has greatly benefited from advances in chest X-ray imaging, which offers fast, cost-effective, and accessible diagnostic capabilities. However, interpreting X-ray images remains challenging due to the visual similarity between pathological features such as opacities and consolidations and normal anatomical structures. This has prompted growing interest in artificial intelligence (AI) and nature-inspired optimization approaches to enhance image segmentation and classification accuracy.

Several recent studies have employed metaheuristic algorithms to improve threshold-based image segmentation. Das et al. [18] proposed a novel non-entropy-based multilevel thresholding approach for COVID-19 chest X-ray image segmentation, utilizing a segmentation score (SS) as the fitness function combined with a chance-based birds’ intelligence optimization algorithm. Their method demonstrated superior performance over traditional entropy-based techniques, achieving significant improvements in image quality metrics such as PSNR, SSIM, and FSIM, thereby enhancing lesion localization and segmentation precision. Otair et al. [19] developed an adapted arithmetic optimization algorithm (AOA) to enhance multilevel thresholding in chest X-ray images, demonstrating strong accuracy and computational efficiency. Similarly, Lan and Wang [20] proposed OLAVOA, an improved African vultures optimization algorithm that integrates predatory memory and logarithmic spirals with opposition-based learning. Their method, optimized using 2D Kapur entropy, demonstrated superior convergence and segmentation precision in COVID-19 chest X-rays.

Complementary to segmentation, classification performance has been enhanced using ensemble and deep learning techniques. Sinra and Angriani [21] applied ensemble machine learning methods for COVID-19 detection in chest X-rays, achieving high predictive performance. Thamilarasi et al. [22] proposed an ensemble segmentation approach combining denoising autoencoders with CLAHE-based preprocessing, which significantly improved image clarity and diagnostic accuracy.

In the broader context of deep learning in chest X-ray interpretation, Ahmad et al. [23] provided a systematic review of machine learning-augmented diagnostic models, highlighting their potential to reduce human error and enhance clinical outcomes. Mathumetha et al. [24] reviewed feature extraction techniques and deep learning frameworks for the early detection of lung nodules, emphasizing the role of automated tools in improving early diagnosis. Kebache et al. [25] developed a CNN-based model for tuberculosis detection, while Suksatan et al. [26] proposed a hybrid transfer learning approach for COVID-19 classification, underscoring the adaptability of deep models in clinical imaging tasks.

Explainable AI (XAI) has also become increasingly important in medical imaging. Sharma et al. [27] embedded XAI within a segmentation-based classification pipeline for COVID-19 detection, improving model transparency. Li et al. [28] developed COVID-MobileXpert, an on-device diagnostic system using chest X-rays for triage and follow-up, further advancing the portability and real-world utility of AI solutions.

Although many existing methods rely on pre-segmented or manually processed data, our work introduces a fully automated framework that integrates Otsu-based multi-level thresholding with nature-inspired optimizers AMO, EMO, and HSA to refine segmentation. This is followed by robust classification using a custom-trained CNN. The proposed method aims to deliver accurate, interpretable, and scalable diagnostic support for lung diseases, particularly in resource-constrained environments (Table 1).

## 3. Materials and Methods

### 3.1. Dataset Characteristics

The X-ray images used for this study were obtained from publicly available Kaggle repositorie, including both original and augmented chest X-rays [31]. The dataset was divided into training, testing, and validation sets to ensure robust model evaluation. The split was performed while maintaining the class distribution (Normal, Pneumonia, COVID-19) across all sets. The distribution of images across the sets is summarized in Table 2.

Table 3 presents the key image specifications following preprocessing. Each image in the dataset was standardized to ensure uniform input for model training.

### 3.2. The Proposed Method

For this purpose, the proposed system begins by processing an X-ray image of a patient’s lungs, resizing it to a uniform resolution of 256 × 256 pixels to maintain consistency between all images for reliable analysis. Contrast adjustment is then applied to improve the visibility of lung structures, enhancing image quality and clarity for more accurate assessment. Following these pre-processing steps, a multilevel segmentation method using the objective Otsu function is employed to effectively separate the lung regions from the background, a key step for accurate diagnosis.

In the next phase, a convolutional neural network model is used during classification [32]. The CNN is specifically trained to classify segmented lung images into three categories: Normal, Pneumonia, and COVID-19. This classification process is integral to the diagnosis of the condition according to the visual patterns present in the radiographic images.

**Figure 1:**
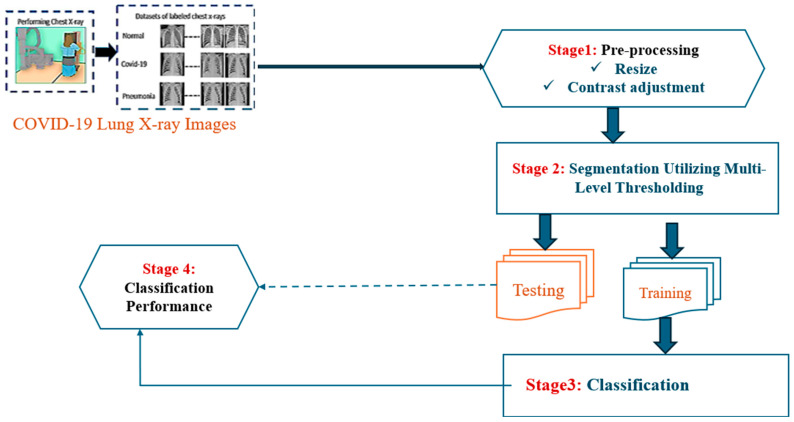
Schematic overview of the proposed system.

In the following sections, the detailed pre-processing, segmentation, and classification procedures are explained in detail. In addition, the complete architectural design of the proposed COVID-19 classification system is depicted below in Figure 1, providing a clear overview of the workflow and interactions between the different components of the system.

#### 3.2.1. Multi-Threshold Segmentation Using Nature-Inspired Optimization Algorithms and Otsu’s Function

##### Animal Migration Optimization Algorithm (AMO)

The AMO algorithm is a population-based optimization technique inspired by the natural behavior of animal swarms during migration, a phenomenon observed in species such as insects, birds, mammals, and reptiles. These migrations, often driven by the search for suitable climates and food resources, are governed by three fundamental behavioral rules: (1) coordinating movement with neighbors, (2) maintaining proximity to neighbors, and (3) avoiding collisions with neighbors. By mimicking these principles, the AMO algorithm effectively solves complex optimization problems. The algorithm operates through two main processes: migration of individuals, where solutions move to new positions while adhering to the three rules, and population update, where underperforming individuals are replaced to maintain population size. Together, these processes enable AMO to explore the search space efficiently and converge toward optimal solutions.

Like other optimization algorithms, AMO begins by randomly initializing the initial positions of the population [33]. Let NP be the size of the population and let D be the dimension of the problem. Initialization of the i-th individual is performed in the following way:

(1) $${x\;}_{d,i,0}=x_{d,l}+{rand}_{i,d}\times (x_{d,u}-x_{d,l})$$

**Figure 2:**
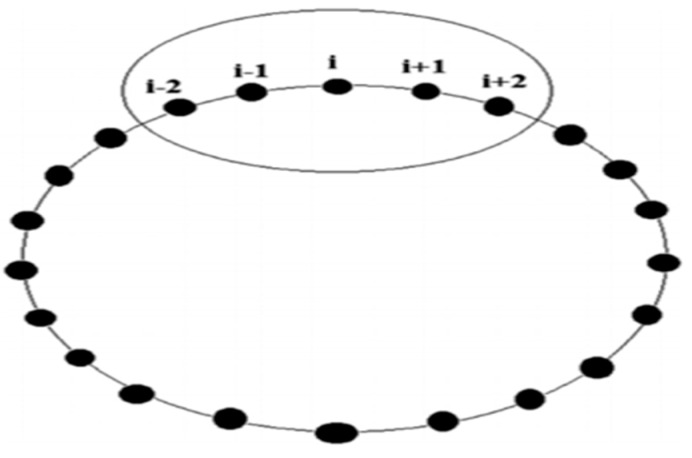
An individual’s local neighborhood scheme in the AMO algorithm.

Here, *d* = 1, ……, D and *i* = 1, ……, NP, where ${rand}_{i,j}\;$ is a uniformly random number in the interval [0, 1]. Variables l and *u* represent, respectively, the upper and lower boundaries for the optimization problem. Three main rules must be respected in this phase of migration to: (1) move toward neighbors in the same way, (2) stay in close proximity to neighbors, and (3) avoid colliding with neighbors. Then, each individual adjusts its position in relation to its neighbors, while ensuring that all positions remain distinct. In AMO, communication between neighbors uses a ring topology, where the latest index connects to the first, creating a circle. Everyone considers the length of its neighborhood to be five. Therefore, for the i-th individual, the neighbors comprise the indices {i − 2, i − 1, i + 1, i + 2}, as shown in Figure 2.

At each iteration, the neighborhood structure is built for each individual. The individual’s position is then updated based on a randomly selected neighbor. The process is as follows:

(2) $$x_{i,G+1}=x_{i,G}+\delta \times (x_{neighbourhood,G}-x_{i,G})$$

The current position of the randomly selected neighborhood is denoted by $x_{neighborhood,G}$, while δ is a problem-related parameter that is generated randomly from a Gaussian distribution.

The migration process involves selecting certain individuals to be removed from the swarm and replacing them with new ones, ensuring that the population size remains constant. During this update, decisions are made about which individuals to eliminate and which to introduce, based on probability *pa*. This probability is determined by the fitness value of each individual: the highest-performing individual is given a *pa* value of 1, while the least fit individual receives a *pa* value of 1/NP, where NP represents the population size. The mechanism for updating the population can be mathematically represented using inference rules, as illustrated below:

(3) $$\frac{\begin{array}{c} p\\ p\to q \end{array}}{:.q}$$

Let the initial assertion be represented as:

- *p*: rand [0, 1] > *pa* and the final assertion,
- *q:* temp = $x_{i,G}$+ δ × ($x_{best,G}-x_{i,G}$) + rand × ($x_{r2,G}+x_{i,G})$.

The premises of the argument include *p → q*, where *q* serves as the conclusion. The indices $r_{1}$ and $r_{2}$ are randomly selected integers within the range [1, NP], with the condition that $r_{1}$ ≠ $r_{2}$. Following this, the new solution for the generation after the *G-th* iteration is chosen between $x_{i,G}$ and temp, depending on which demonstrates better fitness.

**Figure 3:**
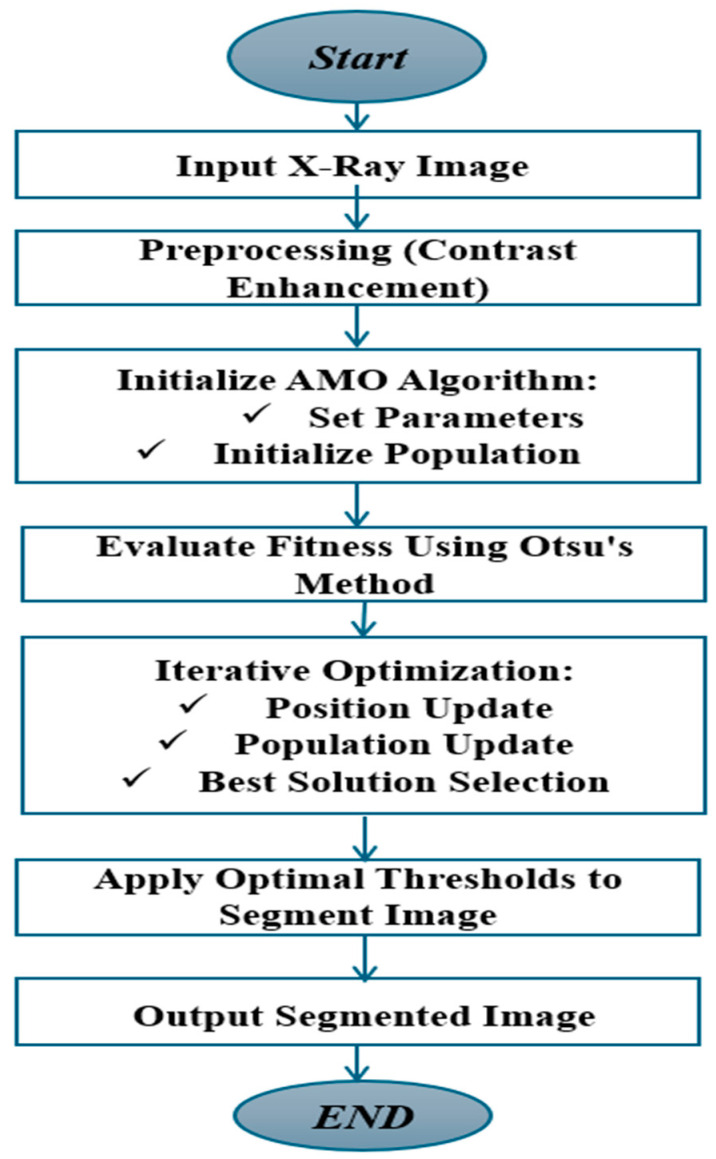
AMO algorithm.

The AMO algorithm is effectively applied to COVID-19 lung image segmentation through a structured and iterative process. By optimizing threshold values using Otsu’s method, the algorithm achieves precise segmentation of infected regions, healthy tissue, and background. The flowchart (Figure 3) provides a clear visual representation of this process, highlighting the key steps from preprocessing to final segmentation. This approach ensures accurate and robust results, making the AMO algorithm a valuable tool for diagnosing and analyzing COVID-19 lung images.

In our segmentation pipeline, the AMO algorithm is used to determine the optimal set of intensity thresholds that segment chest X-ray images into diagnostically relevant regions. Each individual in the AMO population encodes a vector of threshold values, and the fitness of each candidate is computed using Otsu’s between-class variance function. The migration dynamics of AMO, which mimic animal group behavior, enable the algorithm to explore the solution space effectively while avoiding premature convergence. This is particularly important in medical images, where subtle grayscale differences can define pathological boundaries. By applying AMO in this context, the algorithm dynamically adapts threshold vectors over generations to enhance region contrast, resulting in clearer segmentation of lung zones affected by COVID-19 and pneumonia.

##### Electromagnetism Like Optimization Algorithm

The EMO method is designed to address the challenge of finding a global solution for a nonlinear optimization problem with box constraints, which is defined as follows:

(4) $$\mathrm{Maximize}\;f(x),\;\;\;\;\;\;\;\;\;\;\;\;x=(x_{1},\ldots .,x_{n})\;\epsilon R^{n}$$

Subject to                         x ϵX

where f: $R^{n}$ → R is a nonlinear function whereas X = $\left\{x\;\epsilon \;R^{n}|l_{i}\leq x_{i\;}\leq u_{i},\;i=1,\ldots n\right\}$ is a bounded feasible region, constrained by the lower ($l_{i})$ and upper ($u_{i})$ limits.

Electromagnetism optimization (EMO) [34] employs a population of N-dimensional points, $x_{i,t}$, to explore the feasible set X, where *t* represents the iteration (or generation) number. Initially, at *t* = 1, the population S*_t_* = {$x_{1,t}$, $x_{2,t}$, ……… $x_{N,t}$} consists of uniformly distributed samples within the search region X. The population set at the t-th iteration, denoted as S_t_, evolves with each iteration. After initializing S_t_, EMO proceeds iteratively until a stopping criterion, such as reaching the maximum number of iterations, is satisfied. Each iteration of EMO consists of two main steps. In the first step, each point in S_t_ moves to a new position based on the attraction–repulsion mechanism, which is derived from electromagnetism theory [35].

In the second step, the points that have been adjusted using the electromagnetism principle undergo an additional local search. These modified points are then incorporated into **S**_t+1_ for the (t + 1)-th iteration. Both the attraction–repulsion mechanism and the local search play critical roles in EMO, guiding the points *x_i,t_* toward the vicinity of the global optimum. Similar to electromagnetism theory for charged particles, each point *x_i,t_* ∈ S_t_ in the search space X is treated as a charged particle, with the charge corresponding to its objective function value. Points with better objective function values are assigned higher charges, resulting in a stronger attraction to other points in S_t_, while points with lower charges repel others. This attraction–repulsion mechanism ensures that points with higher charges attract others, while those with lower charges induce repulsion.

Finally, the total force vector $F_{i}^{t}$ acting on a point, such as the i-th point *x_i,t_*, is calculated by summing the attraction and repulsion forces. Each point *x_i,_*_t_ ∈ **S**_t_ is then relocated toward the new position based on its total force *y_i,t_*. A local search is then performed around each *y_i,t_* to refine its position to *z_i,t_*. The updated points *x_i,t+_*_1_ ∈ S_t+1_ of the (*t* + 1)-th points for the (*t* + 1)-th iterations are established through this process.

(5) $$x_{i,\;\;t+1}=\left\{\begin{array}{c} y_{i,t\;\;\;\;\;\;\;if\;\;\;\;f\;\left(y_{i,\;t}\;\right)\leq \;\;f\left(z_{i,\;t}\right)}\\ z_{i,\;t\;\;\;\;\;\;\;\;\;\;\;\;\;\;\;\;\;\;\;\;\;\;\;\;\;\;\;\;otherwise} \end{array}\right.$$

The electromagnetism-like optimization (EMO) algorithm is a population-based optimization technique inspired by electromagnetism theory. It solves nonlinear optimization problems with box constraints, making it suitable for medical image segmentation, particularly for COVID-19 lung images.

The key steps of EMO for X-ray lung image segmentation focus on:

(a) **Initialization**:
  - Initialize a population of points uniformly within the search space.
  - Each point represents a potential set of threshold values for segmenting the X-ray lung image.
  - Evaluate the fitness of each point using the objective function (Otsu’s method) to measure segmentation quality.
(b) **Iterative Optimization**:
  - Apply the attraction–repulsion mechanism: Points with higher fitness (better segmentation results) attract others, while points with lower fitness repel them, guiding the population toward optimal threshold values.
  - Perform a local search around each point to refine its position, ensuring precise segmentation of lung regions (e.g., infected areas, healthy tissue).
  - Update the population for the next iteration based on the refined positions.
(c) **Termination**:
  - Stop the algorithm when a stopping criterion (e.g., maximum iterations) is met.
  - Select the best set of threshold values for segmenting the X-ray lung image.
  - Generate the final segmented image.

In our segmentation framework, electromagnetism-like optimization is applied to refine the selection of multiple threshold values for X-ray image segmentation. Each candidate solution is modeled as a charged particle in a simulated electromagnetic field, and the fitness based on Otsu’s between-class variance determines whether particles attract or repel each other. This mechanism promotes diverse exploration of the search space while guiding convergence toward globally optimal threshold combinations. EMO’s key advantage is its ability to dynamically balance exploration and exploitation, which is particularly effective for identifying meaningful grayscale intensity boundaries in noisy or low-contrast medical images. Consequently, EMO improves segmentation reliability and produces well-separated lung regions that support more accurate CNN-based classification.

##### The Harmony Search Method

In the fundamental HSA algorithm, each solution is referred to as a “harmony” and represented as an n-dimensional real vector. The algorithm starts by randomly generating a population of harmony vectors, which are stored in a harmony memory (HM). A new candidate harmony is then created by selecting elements from the HM through a memory consideration operation, which may include random reinitialization or pitch adjustment. The HM is updated by comparing the new candidate with the worst harmony vector in memory. If the new harmony yields a better solution, it replaces the worst vector. This cycle continues until a predefined stopping condition is met. The basic HSA involves three main stages: initializing the HM, improving new harmony vectors, and updating the HM. Detailed explanations of each stage are provided in the following sections.

**Setting Up the Problem and Algorithm Parameters**

Generally, the global optimization problem can be described as follows:

■ Minimize f (x), x = ($x_{1}$, $x_{2}$, …, $x_{n}$) ∈ $R^{n}$,
■ Subject to: $x_{j}$, ∈ [$l_{j}$, $u_{j}$], j = 1, 2, …, n,

where *f*(*x*) is the objective function, x = ($x_{1}$, $x_{2}$, …, $x_{n}$) represents the set of design variables, *n* is the number of design variables, and $l_{j}$ and $u_{j}$ are the lower and upper bounds for the design variable $x_{j}$, respectively. The harmony search algorithm (HSA) [36] involves several important parameters: the harmony memory (HM) size, which specifies the number of solution vectors stored; the harmony memory consideration rate (HMCR); the pitch adjustment rate (PAR); the distance bandwidth (BW); and the number of improvisations (NI), which represents the total number of iterations. The effectiveness of HSA is largely determined by the values assigned to these parameters, which can differ based on the particular application.
**Initialization of Harmony Memory**

In this phase, the components of the initial harmony memory (HM) are configured with HMS vectors. Each harmony vector xi = {$x_{i}(1)$, $x_{i}(2)$, …, $x_{i}(n)$,} is generated randomly, where:

(6) $$x_{i}(j),=l(j)++(u(j)\;-\;l(j)).\;rand\;(0,1)$$

for j = 1, 2, …, n and i = 1, 2, …, HMS. Here, rand (0,1) denotes a random value uniformly distributed between 0 and 1, and *l*(*j*) and *u*(*j*) represent the lower and upper bounds of the search space, respectively. The HM matrix is then filled with the HMS harmony vectors in the following manner:

(7) $$\mathrm{HM}=\left[\begin{array}{c} \begin{array}{c} x_{1}\\ x_{2} \end{array}\\ .\\ .\\ .\\ x_{HMS} \end{array}\right]$$

**Generation of New Harmony Vectors**

In this phase, a new harmony vector $x_{new}$ is generated by applying three operations: memory consideration, random reinitialization, and pitch adjustment. This process of generating a new harmony is referred to as “improvisation”. During the memory consideration step, the value of the first decision variable $x_{new}\left(1\right)$ for the new vector is randomly chosen from the values already present in the current harmony memory (HM), specifically from the set {$x_{1}(1)$, $x_{2}$(1), …, $x_{HMS}$ (1)}.

$To\;determine\;how\;to\;generate\;x_{new}(1)$, a uniform random number $r_{1}$ is generated within the range [0, 1]. If $r_{1}$ is less than the harmony memory consideration rate (HMCR), the decision variable $x_{new}\left(1\right)\;is\;selected\;through\;memory\;consideration$, ${\;Otherwise,x}_{new}(1)$ is randomly reinitialized within the search bounds [*l* (1), *u*(1)]. The other decision variables $x_{new}\left(2\right),\;x_{new}\left(3\right),\ldots ..,x_{new}\left(n\right)\;$ are chosen in a similar manner.

Both memory consideration and random reinitialization can be expressed as follows:

(8) $$x_{new}\left(j\right)=\left\{\begin{array}{c} x_{i\;}\left(j\right)\epsilon \;\{x_{1\;}\left(j\right)\;,\;x_{2}\left(j\right),\ldots ..x_{HMS\;}\left(j\right)\;\;\;\;\;\;\;\;\;\;\;\;\;\;\;\;\;\;with\;probability\;HMCR\\ l\left(j\right)+\left(u\left(j\right)-l\left(j\right)\right).rand\;\left(0,1\right)\;\;\;\;\;\;\;\;\;\;\;\;\;\;\;\;with\;\;probabilty\;1-HMCR \end{array}\right.$$

where *j* = 1, 2, …, *n*. This process ensures that the new harmony vector incorporates both existing solutions from the harmony memory and newly generated values within the defined search bounds.

Each component chosen through memory consideration is subsequently assessed to determine whether it should undergo pitch adjustment. The pitch adjustment rate (PAR) defines how often these adjustments occur, while the bandwidth factor (BW) controls the range of the local search around the selected elements in the harmony memory (HM). The decision to perform a pitch adjustment is based on the following criteria:

(9) $$x_{new}\left(j\right)=\left\{\begin{array}{c} \;\;x_{new}\left(j\right)=x_{new}\left(j\right)\pm rand\left(0,1\right).\;BW,\;\;\;\;with\;probability\;\;PAR\\ x_{new}\left(j\right)\;\;\;\;\;\;\;\;\;\;\;\;\;\;\;\;\;\;\;\;\;\;\;\;\;\;\;\;\;\;\;\;\;\;\;\;\;\;\;\;,\;\;\;\;\;with\;\;probabilty\;1-PAR \end{array}\right.$$

Pitch adjustment is a process that creates new potential harmonies by making small modifications to the original variable positions. This operation is analogous to the mutation process in evolutionary algorithms. Specifically, a decision variable is either altered by adding a random number within the range of 0 and BW or remains unchanged. To ensure the validity of the pitch adjustment, it is crucial to verify that the adjusted points remain within the feasible range [*l*, *u*]. Any points that fall outside this interval must be reassigned to the nearest boundary values, either the minimum or maximum of the range.

**Updating the Harmony Memory Matrix**

After a new harmony vector $x_{new}\;$ is generated, the harmony memory is updated through a competitive process based on fitness, comparing $x_{new}\;with\;the\;worst\;harmony\;vector\;x_{w}$ in the HM. If the fitness value of $x_{new}\;is\;better\;than\;that\;of\;x_{w}$, then $x_{new}\;$ replaces $x_{w}$ and is added to the harmony memory.

**Computational Procedure**

The fundamental steps of the harmony search algorithm (HSA) are presented in Algorithm 1 [37], which is commonly applied to minimization problems. If the goal is to maximize the objective function, step 4 must be adjusted so that $x_{w}$ is replaced by $x_{new}\;$ when $f(x_{new})\;$> ${f(x}_{w})$. In this study, the HSA is employed for maximization purposes.

The harmony search algorithm (HSA) is a metaheuristic optimization technique inspired by musical improvisation. It is applied to X-ray lung image segmentation to optimize threshold values for accurately separating infected regions from healthy tissue.

The key steps are based on:

(a) **Initialization**:
  - Define parameters: harmony memory size (HMS), harmony memory considering rate (HMCR).
  - Pitch adjustment rate (PAR), bandwidth (BW), and number of improvisations (NI).
  - Initialize the harmony memory (HM) with random harmonies (threshold sets) and evaluate their fitness using the objective function (Otsu’s method).
(b) **Improvisation**:
  - Generate a new harmony by:
    - Selecting values from HM (if r_1_ < HMCR).
    - Adjusting values using pitch adjustment (r_2_ < PAR).
    - Generating random values within bounds (if r_1_ < ≥ HMCR).
(c) **Update HM**:
  - Replace the worst harmony in HM with the new harmony if it improves fitness.
(d) **Termination**:
  - Repeat until NI is reached.
  - Return the best harmony (optimal thresholds) for segmentation.
  - HSA optimizes thresholds to segment infected regions and healthy tissue in X-ray lung images.

##### Thresholding in Image Processing

Thresholding is a method used to classify the pixels of a grayscale image into distinct groups or classes based on their intensity levels (L) [38]. This classification requires selecting a threshold value (*th*), and the grouping is performed following the straightforward rule described in Equation (17):

(10) C1←p   if 0≤p<th C2←p  if th≤p≤L−1

Here, *p* represents one of the m × n pixels of the grayscale image I_g_, which can be expressed in grayscale levels L = {0, 1, 2, …, L − 1}. *C*1 and *C*2 are the classes where the pixel p may belong, with *th* serving as the threshold. The rule in Equation (17) applies to bi-level thresholding but can be easily generalized for multiple classes, as demonstrated in Equation (11):

(11) $$\left\{\begin{array}{c} .\;\;\;\\ \begin{array}{c} \;\;C1\leftarrow p\;\;\;\;\;\;\;\;\;\;\;if\;0\leq p<{th}_{1}\;\\ \;C2\leftarrow p\;\;\;\;\;\;\;\;\;\;if\;\;{th}_{1}\leq p\leq {th}_{2}\\ \;\;\;\;.\;\;\;\;\;\;\;\;\;\;\;\;\;\;\;\;\;\;\;\;\;\;\;\;\;\;\;\;\;\;\;\;\;\;\;\;\;\;\;\;\;\;\;\;\;\;\;\;\;\;\;\;\\ \;\;.\;\;\;\;\;\;\;\;\;\;\;\;\;\;\;\;\;\;\;\;\;\;\;\;\;\;\;\;\;\;\;\;\;\;\;\;\;\;\;\;\;\;\;\;\;\;\;\;\;\;\;\;\;\;\;\\ \;\;.\;\;\;\;\;\;\;\;\;\;\;\;\;\;\;\;\;\;\;\;\;\;\;\;\;\;\;\;\;\;\;\;\;\;\;\;\;\;\;\;\;\;\;\;\;\;\;\;\;\;\;\;\;\;\;\\ Ci\leftarrow p\;\;\;\;\;\;\;\;\;\;\;\;\;if\;{th}_{i}\leq p\leq {th}_{i+1}\\ \;Cn\leftarrow p\;\;\;\;\;\;\;\;\;if\;{th}_{n}\leq p\leq L-1 \end{array} \end{array}\right.$$

where ${th}_{1}$, ${th}_{2},\;\ldots ..\;,{th}_{i},\;{th}_{i+1}\;\mathrm{and}\;{th}_{n}$ represent the various threshold values. The main challenge in both bi-level and multilevel thresholding lies in determining the appropriate th values to accurately distinguish the classes.

In our intelligent algorithms, Otsu’s method is utilized as the fitness function. This well-known approach is effective for determining optimal threshold values by proposing objective functions that need to be maximized. By incorporating Otsu’s method, our algorithms can accurately identify threshold values, thereby enhancing the classification and segmentation of pixels in grayscale images. This integration ensures that the algorithms perform optimally, achieving high precision in image processing tasks.

In the context of our multi-threshold segmentation task, the harmony search algorithm is implemented to generate and evolve sets of threshold values that maximize Otsu’s objective function. Each harmony corresponds to a threshold vector, and new harmonies are created by combining values from the existing harmony memory or introducing small stochastic adjustments. This strategy balances the exploration of new regions in the search space with the exploitation of known high-performing solutions. HSA is especially well suited to medical image segmentation because it does not require gradient information and can efficiently adapt to the complex, non-convex landscape presented by grayscale lung X-ray histograms. Through this application, HSA improves the delineation of anatomical structures and pathological regions, producing segmented outputs that are more consistent and clinically meaningful.

##### Between-Class Variance (Otsu’s Method)

Otsu’s method [39] is a nonparametric technique for thresholding that employs the maximum variance value of different classes as a criterion to segment an image [40]. Taking the L intensity levels from an intensity image or from each component of a red, green, and blue (RGB) image, the probability distribution of the intensity values is computed as follows:

(12) $$P_{h_{ci}}=\frac{h_{ci}}{NP};\;\sum_{i=1}^{NP}P_{h_{ci}}=1;\;c=c=\left\{\begin{array}{c} 1,2,3\;\;\;\;\;\;\;\;\;\;\;\;\;if\;\;\;RGB\;image\\ 1\;\;\;\;\;\;\;\;\;\;\;\;\;\;\;\;\;\;\;\;\;\;\;\;if\;grayscale\;image \end{array}\right.$$

Here, i denotes a specific intensity level (0 ≤ i < L); c refers to the image component, which depends on whether the image is grayscale or RGB; and NP is the total number of pixels in the image. *h_ci_* (the histogram) represents the count of pixels corresponding to the intensity level i in component c. The histogram is normalized to form a probability distribution P_hci_.

In the case of the simplest segmentation (bi-level), the image is divided into two classes, which are defined as follows:

(13) $$C1=\{P_{h_{c1}},\ldots ,\;P_{h_{c_{th}}}\}/w_{c}^{0}\left(th\right)$$

(14) $$C2=\{P_{h_{c_{th+1}}},\ldots ,\;P_{h_{c_{L}}}\}/w_{c}^{1}(th)$$

where $w_{c}^{0}(th)$ and $w_{c}^{1}(th)$ are the probability distributions for C1 and C2, as shown by:

(15) $$w_{c}^{0}(th)=\sum_{i=1}^{th}P_{h_{ci\;}};\;w_{c}^{1}(th)=\sum_{i=th+1}^{L}P_{h_{ci\;}}$$

Calculating the mean intensity levels is essential. $\mu_{c}^{0}$ and $\mu_{c}^{1}$ define the classes using:

(16) $$\mu_{c}^{0}=\frac{\sum_{i=1}^{th}iP_{h_{ci\;}}}{w_{c}^{0}(th)};\;\;\;\;\;\;\;\;\;\;\;\;\;\;\;\;\;\;\;\;\;\;\;\;\;\;\mu_{c}^{1}=\frac{\sum_{i=th+1}^{L}{iP}_{h_{ci\;}}}{w_{c}^{1}(th)}$$

Once these values are calculated, the Otsu-based between-class variance $\sigma_{B}^{2}$ is calculated using the following equation:

(17) $$\sigma_{B}^{2}=w_{c}^{0}(\mu_{c}^{0}-\mu_{c}^{T}{)}^{2}+w_{c}^{1}(\mu_{c}^{1}-\mu_{c}^{T}{)}^{2}$$

where $\mu_{c}^{T}$ = $w_{c}^{0}\;\mu_{c}^{0}$ + $w_{c}^{1}\;\mu_{c}^{1}$ and $w_{c}^{0}$ + $w_{c}^{1}$ = 1 Based on the values of $\sigma_{1}^{2}$ and $\sigma_{2}^{2}$, the objective function is presented as follows:

(18) $$f_{Otsu}(\mathrm{th})=\max(\sigma_{B}^{2}(th));\;\;\;\;\;\;\;\;\;\;\;\;\;\;\;0\leq th\leq L$$

The optimization problem is thus reduced to find the intensity level (*th*) that maximizes $\sigma_{B}^{2}(th)$.

Otsu’s method operates on a single component of an image. For RGB images, this requires splitting the image into individual component images. The bi-level thresholding approach described earlier can be extended to determine multiple thresholds. With *k* thresholds, the original image can be divided into *k* classes using Equation (9). The objective function f_Otsu_(*th*) can then be reformulated for multiple thresholds as follows:

(19) $$f_{Otsu}(\mathrm{TH})=\max(\sigma_{B}^{2}(TH));\;\;\;\;\;\;\;\;\;\;\;\;\;\;\;\;\;\;\;\;\;\;\;\;\;\;\;\;\;\;\;\;\;\;\;\;\;\;\;\;\;\;0\;\leq {th}_{i}\leq L;\;\;\;\;\;\;\;\;\;\;i=1,2,\ldots ,k.$$

where TH = [${th}_{1},{th}_{2},\ldots ,{th}_{k-1}$] is a vector containing multiple thresholds and the variances are computed through:

(20) $$\sigma_{B}^{2}=\sum_{i=1}^{k}{w_{c}^{i}(\mu_{c}^{i}-\mu_{c}^{T})}^{2}$$

Here, *i* represents a specific class. $w_{c}^{i}$ and $\mu_{c}^{j}$ are the probability of occurrence and the mean of a class, respectively. For multiple thresholds, these values are obtained as follows:

(21) $$w_{c}^{0}(th)=\sum_{i=1}^{{th}_{1}}P_{h_{ci\;}};\;w_{c}^{1}(th)=\sum_{i={th}_{1}+1}^{{th}_{2}}P_{h_{ci}};\;\;\;\;\;\;\;\;\;\;\;w_{c}^{k-1}(th)=\sum_{i={th}_{k}+1}^{L}P_{h_{ci\;}}$$

And the mean values are obtained as follows:

(22) $$\mu_{c}^{0}=\frac{\sum_{i=1}^{{th}_{1}}iP_{h_{ci\;}}}{w_{c}^{0}({th}_{1})};\;\;\;\;\;\;\;\;\;\;\;\;\;\;\;\;\mu_{c}^{1}=\frac{\sum_{i={th}_{1}+1}^{{th}_{2}}{iP}_{h_{ci\;}}}{w_{c}^{1}({th}_{2})};\;\;\;\;\;\;\;\;\;\;\;\;\;\;\;;\;\mu_{c}^{k-1}=\frac{\sum_{i={th}_{k}+1}^{L}{iP}_{h_{ci\;}}}{w_{c}^{1}({th}_{k})};$$

Like the bi-level scenario, in the case of multiple thresholds using Otsu’s method, c represents the image components: RGB (*c* = 1, 2, 3) or intensity (*c* = 1).

In this study, Otsu’s method serves as the core objective function for identifying optimal thresholds in the image segmentation stage. Its strength lies in maximizing the between-class variance to effectively distinguish anatomical structures in grayscale chest X-ray images. However, when extended to multi-level thresholding, the traditional Otsu’s method becomes computationally expensive and prone to suboptimal solutions due to its exhaustive search strategy. To address these challenges, we integrate Otsu’s criterion with metaheuristic optimization algorithms, namely, the animal migration optimization, electromagnetism-like optimization, and the harmony search algorithm. These algorithms treat the Otsu objective as a fitness function and navigate the high-dimensional threshold space more efficiently and robustly. This hybrid approach significantly improves segmentation performance by avoiding local optima and enabling the extraction of more accurate and diagnostically relevant lung regions. The resulting segmented images serve as high-quality inputs to the subsequent CNN-based classification model, thereby enhancing the overall diagnostic accuracy of the proposed system.

#### 3.2.2. Classification: Proposed Convolutional Neural Network ( ) Model for Lung X-Ray Classification

**Figure 4:**
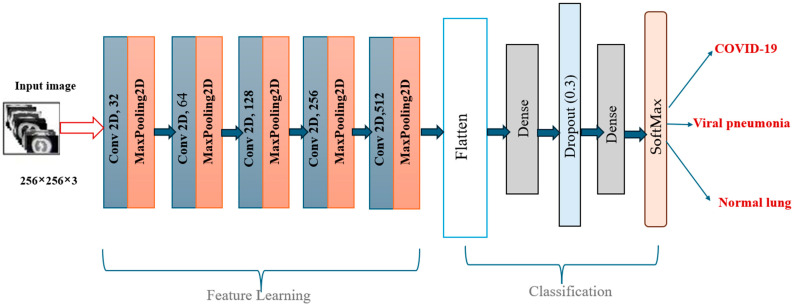
CNN model architecture for lung X-ray image classification.

The proposed CNN model is designed to classify lung X-ray images into three categories: COVID-19 lung, pneumonia lung, and normal lung. This model employs sequential architecture (Figure 4) and processes grayscale images resized to 256 × 256 pixels. It features five convolutional layers with increasing filter sizes (32, 64, 128, 256, and 512) and ReLU activation functions. Each convolutional layer is followed by max-pooling layers to reduce spatial dimensions while retaining essential features (Table 4). A flattening layer is then used to convert the 3D feature maps into a 1D vector. This flattened vector is passed through a fully connected dense layer with 512 units and ReLU activation. To reduce overfitting, a dropout layer with a 0.5 dropout rate is included. The final output layer consists of three units with softmax activation, providing class probabilities for COVID-19 lung, pneumonia lung, and normal lung.

The model is configured using the Adam optimizer with a learning rate of 0.001 and employs categorical cross-entropy as the loss function, while accuracy serves as the evaluation metric. To enhance the model’s robustness, data augmentation techniques such as rotation, shifting, shearing, zooming, and horizontal flipping are applied to the training images. Additionally, early stopping and a learning rate scheduler are implemented to monitor validation accuracy, dynamically adjust the learning rate during training, and prevent overfitting, ensuring efficient and stable model convergence.

Rather than relying on pre-trained architectures such as ResNet or VGG, we opted to develop a custom convolutional neural network tailored specifically for classifying lung conditions in X-ray images. This decision was motivated by the need for a lightweight, fully trainable model that integrates seamlessly with the segmentation outputs produced by our optimization-based thresholding methods. The proposed CNN includes five convolutional layers with increasing filter depth (32 to 512), interleaved with max-pooling operations to progressively reduce spatial dimensions. A fully connected layer with dropout is used to prevent overfitting, followed by a softmax output layer for multi-class classification. This architecture contains approximately 11 million trainable parameters, allowing it to effectively learn relevant spatial patterns without the overparameterization typical of transfer learning models. Although pre-trained models were considered, our experiments showed that the custom CNN achieved comparable accuracy with greater training efficiency and lower inference cost, supporting its suitability for practical deployment in clinical and resource-limited settings.

## 4. Results and Discussion

The proposed method for multi-level thresholding segmentation and classification of lung X-ray images was implemented and tested on a Windows 11 system with an Intel Core i5-4710MQ CPU (2.5 GHz, 8 CPUs), 8 GB of RAM, and 1 GB of dedicated GPU memory. Both the segmentation and classification models were developed using MATLAB R2020a and Python3.11. The performance of the model was assessed using several evaluation metrics, including accuracy, precision, recall, F1-score, and the effectiveness of the optimization algorithms used during the segmentation process.

### 4.1. Metrics for Performance Evaluation

When evaluating the performance of an intelligent algorithm model, especially for tasks like image segmentation and classification, various metrics are commonly used. Each metric provides unique insights into the model’s performance [41]. Below is an overview of the key evaluation metrics that are particularly relevant to this study, focusing on the segmentation and classification of lung X-ray images (COVID-19, pneumonia, and normal).

#### 4.1.1. Accuracy

Accuracy is the ratio of correctly predicted instances (including both true positives and true negatives) to the total number of predictions made.

(23) $$Accuracy=\frac{TP+TN}{TP+TN+FP+FN}$$

#### 4.1.2. Precision

Precision is the ratio of true positive predictions to the total number of positive predictions, which includes both true positives and false positives.

(24) $$\mathrm{Precision}=\frac{TP}{TP+FP}$$

#### 4.1.3. Recall (Sensitivity)

Recall measures the ratio of actual positive instances (true positives) that are correctly identified by the model out of all the actual positives in the dataset.

(25) $$Recall=\frac{TP}{TP+FN}$$

#### 4.1.4. F1-Score

The F1-score is the harmonic mean of precision and recall, offering a balanced measure that combines both metrics to evaluate the model’s performance.

(26) $$F1\_Score=\frac{2\;\times \left(precision\;\times \;recall\right)}{\left(precision+recall\right)}$$

#### 4.1.5. Specificity

Specificity measures the ratio of actual negative cases (true negatives) that are correctly identified by the model out of all the actual negatives in the dataset.

(27) $$Specificity=\frac{TN}{TN+FP}$$

#### 4.1.6. Dice Similarity Coefficient (DSC)

The Dice similarity coefficient (DSC) is a measure of the overlap between two sets, commonly used in image segmentation to assess how well the predicted segmented region aligns with the ground truth.

(28) $$DSC=\frac{2TP}{\left(FP+TP)+(TP+FN\right)}$$

#### 4.1.7. Jaccard Index (Intersection over Union, IoU)

The Jaccard index quantifies the similarity between the predicted segmentation and the ground truth by calculating the ratio of the intersection to the union of the two sets.

(29) $$Jaccard=\frac{TP}{TP+FP+FN}$$

#### 4.1.8. Confusion Matrix

A confusion matrix is a tabular summary of the model’s predictions, displaying the counts of true positives (TP), false positives (FP), true negatives (TN), and false negatives (FN).

### 4.2. Segmentation Performance

The multi-level thresholding technique used during the segmentation phase played a key role in enhancing the classification model’s accuracy. Segmentation was carried out using three optimization algorithms: AMO, HSA, and EMO. These algorithms helped determine the optimal thresholds for distinguishing various regions within the lung X-ray images.

**Figure 5:**
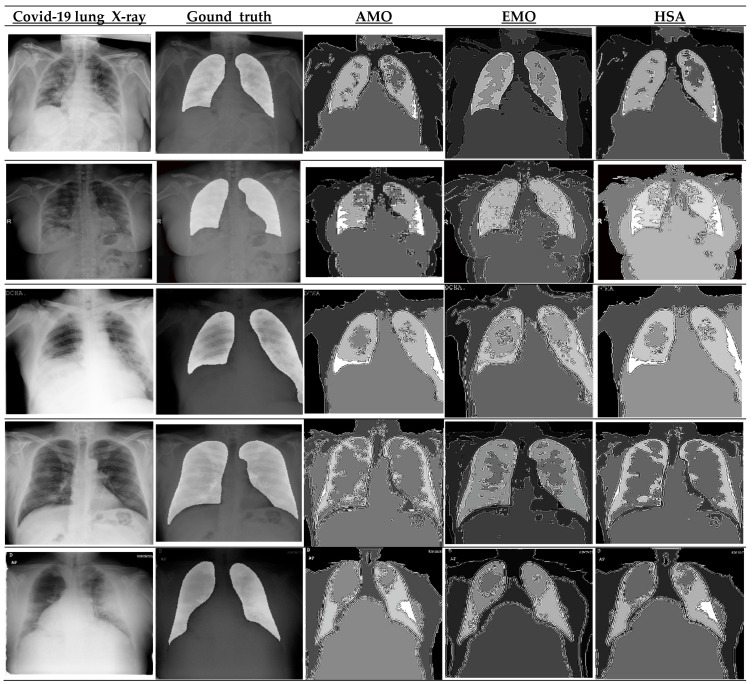
Visual comparison of COVID-19 lung X-ray segmentation results: ground truth vs. AMO, EMO, and HSA algorithms.

Visual inspection of the segmented lung images (Figure 5) demonstrated the superior performance of the EMO algorithm in accurately delineating lung regions, especially in cases with diffuse COVID-19 lesions. EMO consistently outperformed other algorithms, such as AMO and HSA, by capturing subtle differences in pixel intensity and preserving the structural integrity of the lung regions. This precision is crucial for accurately identifying affected areas, aiding in diagnosis and treatment planning. EMO’s robustness in handling complex lung images makes it highly effective for COVID-19 lesion segmentation.

In the discussion of the results obtained from the COVID-19 lung datasets (Table 5), the performance of the three optimization algorithms AMO, EMO, and HAS was evaluated based on accuracy and precision.

To ensure a comprehensive assessment of the segmentation performance of AMO, EMO, and HSA, we constructed multiple test subsets from a COVID-19 chest X-ray dataset. These subsets introduced intra-class variation in terms of lesion density, image contrast, and severity level to mimic real-world diagnostic diversity. This strategy allowed us to evaluate not just raw performance, but also algorithmic robustness across distinct yet clinically relevant imaging scenarios. EMO consistently achieved superior results, reinforcing its reliability for COVID-19 lung segmentation across a range of radiographic conditions.

EMO consistently demonstrated superior performance across all evaluations, achieving the highest accuracy on the chest X-ray dataset with 99.1% ± 0.074 and maintaining strong precision, notably reaching 99.8% ± 0.021. These results highlight EMO’s effectiveness in accurately segmenting and classifying COVID-19 lung images with minimal false positives.

HSA exhibited remarkable precision, particularly on two subsets of the chest X-ray dataset, achieving an outstanding precision of 99.9% ± 0.045. This indicates its strength in reducing misclassifications. However, its accuracy showed more variability, dropping to 90.34% in one subset, suggesting that while HSA reliably identifies true positives, it may underperform in overall classification when presented with more complex variations.

AMO, although performing well in terms of precision, achieving 99.1% ± 0.05 in one dataset, showed fluctuations in accuracy. For example, it recorded only 80.39% ± 0.0687 accuracy on a more diverse subset, indicating challenges in generalizing across complex imaging patterns.

Overall, EMO proved to be the most balanced algorithm, delivering both high accuracy and precision, making it particularly well-suited for COVID-19 lung image segmentation. HSA, with its superior precision, may be preferable in applications where minimizing false positives is critical. By contrast, AMO showed potential but may require further tuning to improve its performance on heterogeneous datasets.

**Figure 6:**
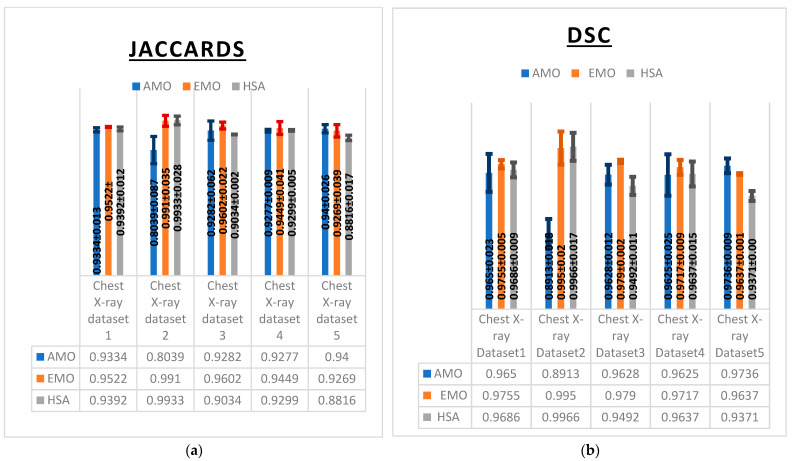
Comparative performance of AMO, EMO, and HSA for COVID-19 lung datasets based on the DSC (**a**) and Jaccard index (**b**).

These observations were further supported by multiple evaluation metrics including the Dice similarity coefficient (DSC), Jaccard index (IoU) (Figure 6), F1-score, specificity, and sensitivity (Table 6) which collectively reflected consistent performance trends across the algorithms AMO, EMO, and HSA.

EMO exhibited superior performance in segmentation accuracy, as reflected by its consistently high Dice similarity coefficient (DSC) values (Figure 6a), achieving 0.995 on Chest X-ray Dataset 2 and 0.979 on Chest X-ray Dataset 3. HSA also demonstrated strong performance, slightly outperforming EMO on Chest X-ray Dataset 2 with a DSC of 0.9966. By contrast, AMO yielded lower DSC values, particularly on Chest X-ray Dataset 2, where it achieved only 0.8913, indicating challenges in segmenting certain image types.

The Jaccard index (IoU) results further confirmed the strength of EMO and HSA (Figure 6b). EMO achieved an impressive 0.9910 on Chest X-ray Dataset 2 and 0.9602 on Chest X-ray Dataset 3, demonstrating its accurate segmentation capabilities. HSA performed comparably, with a value of 0.9933 on Chest X-ray Dataset 2. However, AMO showed a significant drop in IoU on the same dataset, reaching only 0.8039, which reflects its difficulty in accurately identifying lung regions.

The F1-score results also highlighted EMO’s balanced performance, particularly on Chest X-ray Dataset 2, where it achieves 99.55%. HSA followed closely, with strong scores on Chest X-ray Datasets 2 and 3. However, its performance declined slightly on Chest X-ray Dataset 5, where it achieved 93.71%, indicating a reduced ability to handle more complex cases. While AMO achieved a solid F1-score of 96.56% on Chest X-ray Dataset 1, it fell behind the other algorithms on most datasets, particularly on Chest X-ray Dataset 2, where it reached only 90% (Table 6).

In terms of specificity, HSA excelled, achieving 99% on Chest X-ray Datasets 1 and 4, highlighting its effectiveness in minimizing false positives. EMO also performed well in this metric, reaching 99.07% on Chest X-ray Dataset 3. Although AMO maintained acceptable specificity across most datasets, it showed a slight drop on Chest X-ray Dataset 2 with 97%, indicating a relatively higher rate of false positives.

The recall (Sensitivity) results again placed EMO in the lead, achieving 99.1% on Chest X-ray Dataset 2 and 98.35% on Chest X-ray Dataset 1, confirming its high sensitivity in detecting lung abnormalities. HSA performed similarly, achieving 99.3% on Chest X-ray Dataset 2. However, AMO showed a notable decrease in recall on Chest X-ray Dataset 2, with only 80.39%, revealing its limitation in correctly identifying true positive cases in this dataset.

Overall, EMO proved to be the most balanced algorithm, consistently achieving high performance across all metrics and datasets. HSA also demonstrated strong performance, particularly in precision and specificity, but showed slight variability in handling complex cases. AMO, while effective on some datasets, exhibited noticeable performance drops, particularly on Dataset 2, highlighting the need for further optimization to improve its consistency in segmentation and classification tasks.

### 4.3. Performance Evaluation and Comparison with Recent Literature

The proposed methods AMO, EMO, and HSA were evaluated on COVID-19, pneumonia, and normal chest X-ray datasets. The results, summarized in Table 7, demonstrate the comparative performance of these methods against recent state-of-the-art approaches.

The proposed methods AMO, EMO, and HSA demonstrated state-of-the-art performance in classifying COVID-19, pneumonia, and normal chest X-ray images. AMO achieved high precision (99.1%) and competitive recall (98.84%), making it effective in minimizing false positives. EMO and HSA outperformed existing approaches, with near-perfect accuracy (99.1% for EMO and 99.33% for HSA), precision (99.9% for both), and recall (99.1% for EMO and 99.3% for HSA). These results highlight the robustness of EMO and HSA in handling complex classification tasks and their superiority over recent methods like de Moura et al. (2022) [42], Verma et al. (2024) [43], and Chakraborty & Mali (2022) [44].

### 4.4. Classification Performance

**Figure 7:**
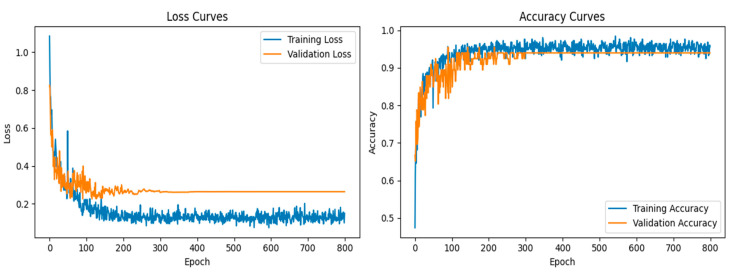
Loss and accuracy curves for training and validation of the CNN model.

In the classification phase, the proposed CNN model exhibited exceptional performance, as demonstrated by the accuracy and loss curves. The accuracy curves showed rapid improvement during the early epochs, with both the training and validation accuracy curves converging toward 95% after approximately 300 epochs (Figure 7). This indicated that the model was able to learn the distinguishing features of the lung X-ray images effectively while maintaining a balance between training and validation performance. The loss curves for both training and validation exhibited steady declines, with minimal overfitting observed, as the gap between the training and validation losses remained narrow throughout the training process.

Although the proposed methodology achieved high performance on a dataset of 306 X-ray images, we acknowledge that this represents a relatively small-scale dataset compared to publicly available repositories like Chest X-ray of COVID-19 lung. The segmentation and classification performance could vary when applied to larger and more heterogeneous datasets. However, the modular structure of our pipeline, including image preprocessing, multi-threshold segmentation, and CNN classification, makes it adaptable for generalization. Future work will involve validating the method on more extensive and diverse datasets to further assess its robustness and scalability in real-world diagnostic settings.

**Figure 8:**
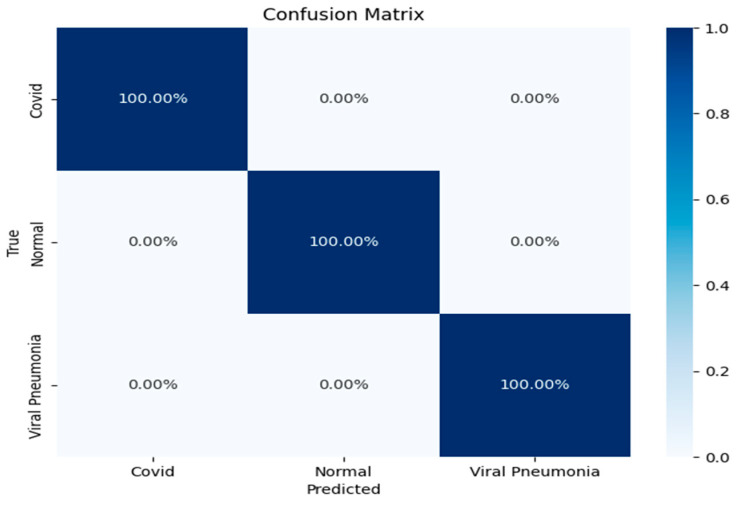
Confusion matrix for the CNN model classification of COVID-19, viral pneumonia, and normal lung X-ray images.

The confusion matrix further reinforced the model’s robustness, showing a perfect classification rate for all three categories (Figure 8): COVID-19, viral pneumonia, and normal lung X-rays. Each of the classes was predicted with 100% accuracy, indicating that the model was able to distinguish between the subtle differences in the lung conditions without any misclassifications. This result underscores the CNN model’s strong generalization ability, especially in a domain where inter-class similarities might challenge less sophisticated models.

Overall, these results highlight the effectiveness of the CNN architecture in accurately classifying lung conditions from X-ray images. The model’s ability to maintain high accuracy across different conditions, coupled with the precise class predictions in the confusion matrix, suggests its potential utility in real-world clinical settings for rapid and reliable diagnosis. These findings demonstrate that the model could significantly enhance diagnostic workflows by providing accurate and automated image-based evaluations, contributing to more efficient disease detection and management.

To understand the source of the model’s high classification performance, we conducted an analysis comparing results with and without the proposed segmentation. The CNN architecture was kept identical in both scenarios. When trained on unsegmented chest X-ray images, the model reached an average accuracy of 89%, indicating moderate classification capability. However, when trained on images pre-processed through our optimized multi-threshold segmentation, the same model achieved accuracy values exceeding 99%. This significant improvement can be attributed to the enhanced clarity and separation of anatomical structures provided by the segmentation. By removing irrelevant background and enhancing the contrast of pathological regions, the segmentation step simplifies the learning task for the CNN. This finding suggests that the strength of our framework lies not in model complexity but in the quality of the image preparation pipeline, which allows even a relatively simple CNN to achieve high precision, recall, and overall accuracy.

The choice of a simple, custom-designed CNN in this study was guided by the goal of evaluating the contribution of the proposed segmentation strategy in a controlled setting. More complex architectures such as ResNet or vision transformers can marginally improve accuracy, but they also introduce considerable computational cost, increased inference time, and complexity, which can obscure the source of performance improvements. By using a lightweight CNN with approximately 11 million parameters, we ensured transparency in the results and demonstrated that high accuracy can be achieved when segmentation quality is maximized. Furthermore, the chosen architecture is more suitable for real-time diagnostic support in clinical environments with limited hardware resources, making it a practical and effective solution for deployment in diverse healthcare settings.

Although our proposed model demonstrated strong classification performance across the dataset, it does not currently distinguish between COVID-19 variants or recognize ARDS specific features, as the dataset lacked such annotations. Different COVID-19 variants tend to affect distinct anatomical regions within the lungs, and ARDS is associated with characteristic radiographic findings that significantly impact clinical outcomes. Future work should incorporate annotated clinical metadata, including variant and ARDS status, to enhance the model’s specificity and clinical utility. Additionally, integrating explainable AI techniques could provide valuable visual insights into the model’s decision-making process by highlighting which lung regions influenced predictions. This would not only improve interpretability but also assist clinicians in verifying that the model aligns with known pathological patterns.

## 5. Conclusions

This study introduces an effective and fully automated approach for segmenting and classifying chest X-ray images to distinguish COVID-19, viral pneumonia, and normal cases. The key contribution lies in combining multi-level Otsu thresholding with three advanced metaheuristic algorithms, AMO, EMO, and HAS, to enhance segmentation accuracy. These optimally segmented images were then classified using a lightweight CNN, achieving high performance across all evaluation metrics.

Among the tested algorithms, EMO demonstrated the most balanced results, contributing significantly to overall classification accuracy, which reached up to 99.1%, with precision and sensitivity above 99%. The integration of optimized segmentation and deep learning not only improved diagnostic reliability but also ensured that the model remained computationally efficient and suitable for deployment in real-time medical settings.

In future work, we plan to expand the dataset to include a wider range of lung conditions and patient demographics, explore domain-adapted pre-trained architectures, and integrate interpretability features to support clinical decision making. This research paves the way for building fast, accurate, and scalable diagnostic tools that can support radiologists in diverse healthcare environments.

## Figures and Tables

**Table 1:** Comparative analysis of recent studies in chest X-ray image diagnosis.

Study	Dataset	Model/Technique	Optimization Algorithm	Performance Metrics
Proposed Method	COVID-19, Pneumonia, Normal (CXR)	CNN + Multi-threshold segmentation	AMO, EMO, HSA	Accuracy up to 99.33%, F1-score 99.6%
Das et al. (2023) [18]	COVID-19 Radiography Database	SS-SGCS (Segmentation Score-based Soft Computing Segmentation)	Chance-based Birds’ Intelligence	PSNR: ~11% improvement over entropy-based methods; Enhanced SSIM and FSIM compared to Tsallis’, Kapur’s, and Masi’s methods.
Otair et al. (2024) [19]	Chest X-ray (5 benchmark images)	AAOA + Multi-thresholding + Histogram	Arithmetic Optimization Algorithm (AAOA)	PSNR: 39.76, SSIM: 0.9873, RMSE: 1.44
Ahmad et al. (2023) [23]	Various Chest X-rays (review)	Systematic Review (ML Augmentation)	N/A	Review of multiple models (no direct performance)
Sinra & Angriani (2024) [21]	COVID-19 Chest X-ray Images	Ensemble Machine Learning (Voting)	None	Accuracy: 95.13%
Mathumetha et al. (2024) [24]	Cancerous Lung Nodules (survey)	Survey of DL/Feature Extraction	None	Survey (no direct metrics)
Kebache et al. (2023) [25]	Chest X-ray (TB classification)	DL for TB Detection	None	Accuracy: 97.89%
Suksatan et al. (2022) [26]	COVID-19 Chest X-ray	Hybrid DL with TL	None	Accuracy: 96.25%
Lan & Wang (2024) [20]	COVID-19 CXR, Brain MRI	Improved AVOA + 2D Kapur	Improved African Vultures Optimization (OLAVOA)	PSNR: 42.38, SSIM: 0.9891 (CXR)
Hage Chehade et al. (2024) [29]	Chest X-ray (multi-disease)	DL Classification Survey	None	Survey
Thamilarasi et al. (2025) [22]	JSRT Chest X-ray	Denoising Autoencoder + CLAHE + Ensemble	None	Accuracy: 97.67%
Saifullah & Dreżewski (2024) [30]	Chest X-ray, Lung CT-scan	PSO + Histogram Equalization	Particle Swarm Optimization (PSO)	Qualitative only (no precise metrics)

**Table 2:** Train–test–validation split.

Dataset	Normal	Pneumonia	COVID-19	Total
Training	70	70	70	210
Testing	20	20	26	66
Validation	10	10	10	30
Total	100	100	106	306

**Table 3:** Dataset distribution and image specifications.

Attribute	Value
Image Format	PNG
Color Mode	Grayscale
Original Resolution	256 × 256 to 1024 × 1024 pixels
Resized Resolution	256 × 256 pixels
Preprocessing Applied	Yes (resizing, contrast enhancement)

**Table 4:** Detailed summary of CNN model architecture for lung X-ray image classification.

Layer (Type)	Output Shape	Parameters
Input Layer	(256, 256, 3)	0
Conv2D (32 filters)	(254, 254, 32)	320
MaxPooling2D	(127, 127, 32)	0
Conv2D (64 filters)	(125, 125, 64)	18,496
MaxPooling2D	(62, 62, 64)	0
Conv2D (128 filters)	(60, 60, 128)	73,856
MaxPooling2D	(30, 30, 128)	0
Conv2D (256 filters)	(28, 28, 256)	295,168
MaxPooling2D	(14, 14, 256)	0
Conv2D (512 filters)	(12, 12, 512)	1,180,160
MaxPooling2D	(6, 6, 512)	0
Flatten	(18,432)	0
Dense (512 units)	(512)	9,437,696
Dropout (0.3)	(512)	0
Dense (3 units–output)	(3)	1539
Total Parameters		11,005,235
Trainable Parameters		11,005,235
Non-trainable Parameters		0

**Table 5:** Comparative performance of AMO, EMO, and HSA for COVID-19 lung datasets based on accuracy and precision.

		Accuracy (%)			Precision (%)		
	Algorithms	AMO	EMO	HSA	AMO	EMO	HSA
Chest X-Ray Datasets							
Chest X-ray dataset 1		93.56 ± 0.0542	95.23 ± 0.058	94.12 ± 0.065	99.1 ± 0.087	96.76 ± 0.054	99.5 ± 0.011
Chest X-ray dataset 2		80.39 ± 0.0687	99.1 ± 0.074	99.33 ± 0.051	99 ± 0.078	99.5 ± 0.068	99.8 ± 0.007
Chest X-ray dataset 3		93.43 ± 0.015	96.37 ± 0.048	90.34 ± 0.28	98 ± 0.087	99.9 ± 0.013	91.12 ± 0.225
Chest X-ray dataset 4		92.84 ± 0.082	94.54 ± 0.089	93.06 ± 0.087	99.1 ± 0.05	99 ± 0.015	99.9 ± 0.045
Chest X-ray dataset 5		94.86 ± 0.038	93 ± 0.056	88.44 ± 0.412	96 ± 0.098	99.8 ± 0.021	99.6 ± 0.020

**Table 6:** Comparative performance of AMO, EMO, and HSA for COVID-19 lung datasets based on recall, specificity and F1-score.

		Recall (Sensitivity) (%)			Specificity (%)			F1-Score (%)			
	Algorithm	AMO	EMO	HSA	AMO	EMO	HSA	AMO	EMO	HSA	
Chest X-Ray Datasets											
Chest X-ray dataset 1		93.34	98.35	93.9	99	95.6	99	96.56	97.55	96.86	
Chest X-ray dataset 2		80.39	99.1	99.3	97	96	90	90	99.55	99.6	
Chest X-ray dataset 3		93.14	96.11	99.06	96.48	99.07	94.3	96.28	97.97	94.92	
Chest X-ray dataset4		92.78	94.49	93	99.03	99.5	99.03	96.25	97.17	96.37	
Chest X-ray dataset 5		98.84	92.79	88.44	91.5	97.55	92.67	97.36	96.21	93.71	

**Table 7:** Performance comparison with recent literature.

Study	Method	Accuracy (%)	Precision (%)	Recall (%)	F1-Score (%)	Dataset
Proposed Method (AMO)	Animal Migration Optimization (AMO)	94.86	99.1	98.84	97.36	COVID-19, Pneumonia, Normal
Proposed Method (EMO)	Electro-magnetism-like Optimization (EMO)	99.1	99.9	99.1	99.55	COVID-19, Pneumonia, Normal
Proposed Method (HSA)	Harmony Search Algorithm (HSA)	99.33	99.9	99.3	99.6	COVID-19, Pneumonia, Normal
de Moura et al. (2022) [42]	Fully automatic deep convolutional approaches	96.5	96.2	96.8	96.5	COVID-19, Pneumonia, Normal
Verma et al. (2024) [43]	Deep learning with histogram equalization and lung segmentation	95.8	95.5	96.0	95.7	COVID-19, Pneumonia, Normal
Chakraborty & Mali (2022) [44]	Super pixel-based fuzzy image segmentation (SUFEMO)	94.7	94.3	95.0	94.6	COVID-19, Pneumonia, Normal
Thamilarasi et al. (2025) [22]	Denoising Autoencoder + CLAHE + Ensemble Segmentation	92.02	-	-		JSRT (Chest X-ray)
Saifullah & Dreżewski (2024) [30]	PSO + Histogram Equalization (HE)	90.65	93.5	93.7	-	Chest X-ray, Lung

## Data Availability

Dataset available on request from the authors.
